# Association of cold weather and influenza infection with stroke: a 22-year time-series analysis

**DOI:** 10.1007/s00484-025-02870-2

**Published:** 2025-03-20

**Authors:** Zihan Yang, Yuchen Wei, Xiaoting Jiang, Conglu Li, Guozhang Lin, Yawen Wang, Ka Chun Chong

**Affiliations:** 1 Jockey Club School of Public Health and Primary Care, Prince of Wales Hospital, The Chinese University of Hong Kong, Shatin, New Territories, Hong Kong Special Administrative Region China; 2 Clinical Trials and Biostatistics Laboratory, Shenzhen Research Institute, The Chinese University of Hong Kong, Shenzhen, China; 3 Centre for Health Systems and Policy Research, The Chinese University of Hong Kong, Ma Liu Shui, New Territories, Hong Kong Special Administrative Region China; 4 Division of Landscape Architecture, Department of Architecture, Faculty of Architecture, The University of Hong Kong, Hong Kong Island, Hong Kong Special Administrative Region China

**Keywords:** Influenza-like illness-positive, Meteorological effect, Cardiovascular disease

## Abstract

Despite the significant adverse health effects and economic burden of stroke inpatients, there has been a paucity of research specifically analyzing and quantifying the association between meteorological factors, air pollutants, influenza infection, and stroke admissions. This study assessed the impact of environmental factors and influenza activity on stroke admissions in Hong Kong from 1998 to 2019, utilizing population-based records. We employed the number of weekly stroke admissions as the primary outcome measure and matched the number with meteorological factors and air pollutant concentrations. Strain-specific influenza-like illness-positive (ILI+) rates were used as a proxy indicator of influenza activity. A quasi-Poisson generalized additive model in combination with a distributed-lag non-linear model (DLNM) was employed to elucidate the relationship of interest. Over the 22-year period, a total of 1,170,882 stroke-related admissions were recorded in Hong Kong. The cumulative adjusted relative risk (ARR) of stroke admission was significantly increased (cumulative ARR = 1.106, 95% CI, 1.069–1.143) at the 5th percentile of temperature (i.e., 15.08 °C) compared with the median reference level. Using zero as the reference value, the cumulative adjusted relative risks of stroke admission were 1.030 (95% CI, 1.018–1.042) at the 95th percentile of ILI + A/H1N1 rate, and 1.022 (95% CI, 1.007–1.038) at the 95th percentile of ILI + total rate. In conclusion, cold weather and influenza infection are risk factors to stroke admissions. Public health strategies for preventing stroke should be considered such as advocating influenza vaccination programmes during winter.

**Supplementary Information:**

The online version contains supplementary material available at 10.1007/s00484-025-02870-2.

## Introduction

Stroke, including ischemic stroke and hemorrhagic stroke, is an acute cerebrovascular disease (Centre for Health Protection of HK 2020; National Heart, Lung, and Blood Institute 2023). Ischemic stroke and hemorrhagic stroke are caused by obstruction of blood flow to the brain and sudden bleeding in the brain, respectively (National Heart, Lung, and Blood Institute 2023). Stroke is the second leading cause of death worldwide and more than 6 million people died from it in 2019, accounting for 11.6% of entire mortalities (GBD 2019 Stroke Collaborators 2021; World Health Organization 2024). From 1990 to 2019, the case number, prevalence, and number of deaths from strokes increased by 70%, 85% and 43%, respectively (Global Burden of Disease Study 2021). In Hong Kong, stroke represents one of the three leading causes of hospitalizations, accounting for the largest proportion of hospital bed days (Woo et al. 2014).

Stroke not only causes destructive physical conditions, but also imposes a severe economic burden on individuals and society. In terms of socio-economic burden, stroke accounts for 34% of total global healthcare expenditure each year (Rochmah et al. 2021). Annual stroke-related expenditures were estimated at $18.8 billion and $6 billion in the United States and China, respectively (Liu et al. 2011; Wang et al. 2014). The global value of lost welfare (VLW) due to stroke in 2019 was approximately $2,056 billion (Gerstl et al. 2023). In terms of personal financial burden, the direct and indirect costs of ischemic stroke for one year were approximately 57,567 CNY (Lv et al. 2024). In the United States and Sweden, the average annual costs for stroke patients were $59,900 and $52,725, respectively (Strilciuc et al. 2021).

Meteorological factors and air pollutants have been shown to be risk factors that could trigger stroke admissions. A cohort study in Japan suggested that lower temperatures and radical changes in humidity were considered risk factors for stroke (Matsumaru et al. 2020). A case-crossover analysis in Canada found that cold temperature and snowfall were independent risk factors of death due to hemorrhagic stroke in men (Polcaro-Pichet et al. 2019). Air pollutants consist of particulate matter (PM) and a variety of toxic gases (Verhoeven et al. 2021). Air pollutants are recognized as a major contributor to the global burden of disease, especially in low-income and developing countries (GBD 2015 Risk Factors Collaborators 2016). Currently, air pollutant is estimated to be responsible for 14% of all stroke-related deaths (Verhoeven et al. 2021). A cohort study and a systematic review indicated that exposure to nitrogen oxides, particulate matter < 2.5 μm (PM_2.5_), particulate matter < 10 μm (PM_10_), and black carbon was associated with increased incidence and mortality of stroke (de Bont et al. 2022; Bont et al. 2023).

The role of influenza infection as a determining factor in the incidence of stroke has been the subject of study for several decades. Infection has been identified as a potential chronic risk factor for stroke and as an acute precipitator (Vollmer et al. 2022; Warren-Gash et al. 2009). Respiratory infections are the most common cause of infection in adults, with influenza-like illness accounting for the majority (Rothman et al. 2006). A case-crossover analysis found that influenza-like illness increases the short-term risk of stroke, particularly in individuals under 45 years of age (Boehme et al. 2018). Furthermore, a case-control study indicated that ILI increased the odds of stroke in young and middle-aged people, while the risk of stroke may be reduced by the vaccination (Vollmer et al. 2022). Hong Kong is an epicentre of influenza, with human influenza A and B viruses being the two main seasonal influenza types circulating locally (Chong et al. 2022). In Hong Kong, influenza A usually has two seasonal peaks in winter/spring and summer each year, while influenza B usually shows a distinct winter/spring peak annually (Chong et al. 2020). Additionally, influenza A infection is associated with more severe illness than influenza B infection (Kim et al. 2009).

Despite the notable incidence of stroke in patients hospitalized with influenza, no previous study has attempted to simultaneously analyze and quantify the relationship between environmental factors, influenza activity and stroke at the population level. The aim of this study was to evaluate the time-varying effects of environmental factors and influenza infection on stroke admissions taking advantage of surveillance data from Hong Kong over a 22-year span. The findings of this study could inform disease management and prevention in stroke patients.

## Methods

### Data sources

#### Hospitalization data

We collected the weekly number of adult (i.e., aged ≥ 18 years) admissions to 41 public hospitals in Hong Kong of the Hong Kong Hospital Authority from January 1, 1998 to December 31, 2019. Stroke records with primary and secondary diagnoses were identified using the International Classification of Diseases (ICD-9-CM) diagnosis code 430–434, which was used as a proxy for stroke hospitalization in this study.

#### Meteorological data

Weekly mean temperature and mean relative humidity were calculated based on daily averages, while total rainfall was quantified based on the sum of weekly precipitation amounts (Li et al. 2023). All these data were obtained from the Hong Kong Observatory to reflect the representative weather conditions in central Hong Kong. Using temperature and relative humidity, we derived the actual vapour pressure, as a proxy of absolute humidity based on the Tetens formula (Tetens 1930). Compared with relative humidity, absolute humidity can be served as a humidity metric, independent to the temperature changes (Chong et al. 2024; Davis et al. 2016).

#### Air pollutant data

Daily averages of air pollutant concentrations including average nitrogen dioxide (NO_2_), sulfur dioxide (SO_2_), ozone (O_3_), and PM_2.5_ were obtained from the monitoring stations of Hong Kong Environmental Protection Department. With the purpose of assessing the combined oxidative capacity of NO_2_ and O_3_ accurately and avoiding collinearity of their combined oxidation effects, the redox-weighted oxidation capacity (O_x_) was used as a proxy (Li et al. 2023; Weichenthal et al. 2017; Xiong et al. 2023). The calculation formula is O_x_ = (1/3) NO_2_ + (2/3) O_3_ (Weichenthal et al. 2017).

#### Influenza data

Influenza-like illness-positive (ILI +) rate from the Hong Kong outpatient sentinel surveillance network was used as a proxy of influenza activity. The ILI + rate was calculated by multiplying the proportion of influenza-like illness consultation by the proportion of virus detection for specific strains (strain A/H1N1, A/H3N2, or B). The ILI + rate represents a more comprehensive and reliable metric of influenza infection because it may reduce part of bias caused by clinical overdiagnosis (Chong et al. 2020).

#### Statistical models

To assess the association between environmental variation, ILI + rate, and stroke hospitalizations, we used a combination of the quasi-Poisson generalized additive model and the distributed-lag non-linear model (Chong et al. 2022; Xiong et al. 2023). This modeling framework enables the simultaneous assessment of exposure-response and lag-response associations (Li et al. 2023; Xiong et al. 2023). The number of weekly hospitalizations due to stroke was set as the response variable, while meteorological factors, air pollutants, and ILI + rate were employed as independent variables of the model. The complete regression model is as follows:

$$log\left(\mu_t\right)=intercept+cb\left(ILI+A/H1N1_t;lag\right)+cb\left(ILI+A/H3N2_t;lag\right)+cb\left(ILI+B_t;lag\right)+cb\left(temp_t;lag\right)+cb\left(humid_t;lag\right)+cb\left(rain_t;lag\right)+cb\left(O_{xt};lag\right)+cb\left(SO_{2t};lag\right)+cb\left(PM_{2.5t};lag\right)+s\left(year_t\right)+s\left(week_t\right)+holiday_t+offset_t+autoregressive\;terms$$

where *µ*_t_ represents the expected number of stroke admissions in week *t*. The terms *ILI + A/H1N1*_*t*_, *ILI + A/H3N2*_*t*_, and *ILI + B*_*t*_ denote the rate of ILI + caused by specific strains in week *t*. The function *cb(.)* denotes the cross-basis function that models both exposure-response relationship and lag-response effects simultaneously (Gasparrini et al. 2010). The function *s(.)* denotes a spline function. Weekly mean air temperature *(temp*_*t*_*)*, relative humidity *(humid*_*t*_*)*, total rainfall *(rain*_*t*_*)*, O_x_ concentration *(O*_*xt*_*)*, SO_2_ concentration *(SO*_*2t*_*)* and PM_2.5_ concentration *(PM*_*2.5t*_*)* were included in the model to assess the effect of environmental exposures. To address the issue of collinearity between air pollutants, the effect of PM_2.5_ was examined separately from O_x_ and SO_2_. As actual vapour pressure is derived by temperature and relative humidity, the effect of actual vapour pressure was examined in a separate model by substituting relative humidity with actual vapour pressure. The term *year*_*t*_ is used to indicate long-term trends of stroke admissions. The term *week*_*t*_ represents the week of year as a proxy for seasonal trends in stroke admissions. *holiday*_*t*_ is a binary variable indicating whether there were public holidays during week *t*. The term offset *(offset*_*t*_*)* is the natural logarithm of the total number of all-caused hospital admissions in week *t*. Autoregressive terms were included in the model to minimize residual autocorrelation (Chong et al. 2020). The degrees of freedom for the exposure variables and the lag parameters in the *cb(.)* functions were determined in a range of two to five by minimizing the generalized cross-validation score, and the maximum lag was set to 2 weeks (Chong et al. 2020). The effects of influenza infection and environmental variables on stroke admissions were quantified by cumulative adjusted relative risks (ARR), which are cumulative adjusted relative risks and their corresponding 95% confidence intervals over the lagged period. The ARR with a 95% CI, excluding unity, was considered statistically significant. The reference values were set as medians for air temperature and relative humidity, zero for total rainfall and ILI + rates, and 5th percentile for air pollutants.

#### Stratified analysis

To examine the modification effect between air pollutant concentration and stroke admission, subgroups were divided based on year, from 1998 to 2010 and from 2011 to 2019.

The signing of the new co-operation arrangement on tackling air pollution by the Environmental Protection Department (EPD) of Hong Kong in 2010 may result in alterations to air pollutant concentrations (News 2010).

All the statistical analyses were performed by executing “dlnm” and “mgcv” packages in R version 4.3.3.

## Results

**Fig. 1:**
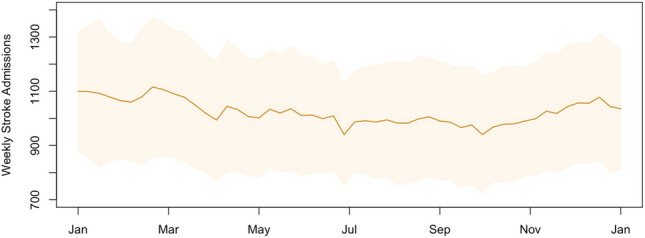
Seasonal trends of stroke admission by week from 1998 to 2019. It is expressed as the mean ± one standard deviation

**Fig. 2:**
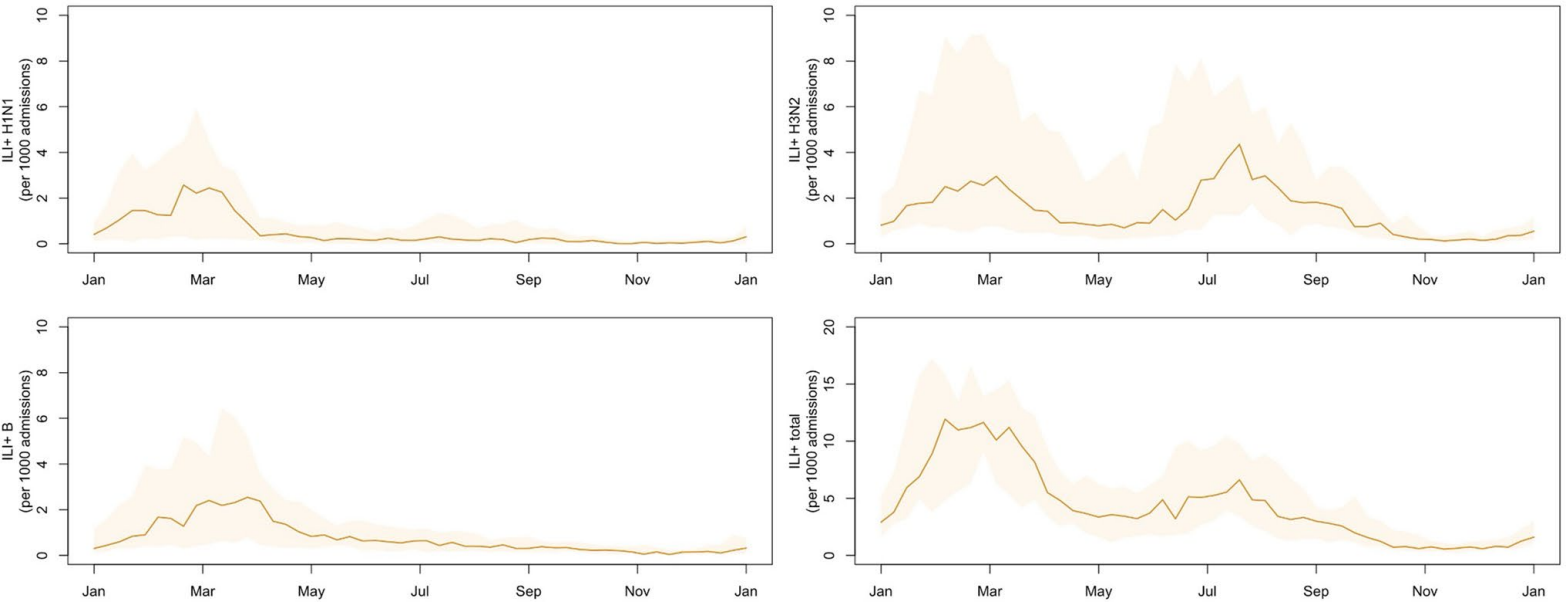
Seasonal trends of ILI + rates by week from 1998 to 2019 They are expressed as median with interquartile range

Over a 22-year period, a total of 1,170,882 stroke-related admissions were recorded in Hong Kong. The temporal distribution of weekly stroke admission counts is shown (Fig. 1). Stroke-related hospitalizations showed a clear seasonality, peaking in winter and plateauing in summer. Descriptive statistics of meteorological variables, air pollutants, and ILI + rates are presented (Table 1). During the study period, weekly mean temperature, relative humidity, and total rainfall altered with medians of 24.61 °C, 79.64%, and 11.35 mm, respectively. Weekly mean O_x_, SO_2_, and PM_2.5_ varied with medians of 42.78 µg/m^3^, 13.15 µg/m^3^, and 30.34 µg/m^3^, respectively. For per 1000 consultations, the median rates (inter-quartile range) of ILI + A/H1N1, ILI + A/H3N2, ILI + B, and ILI + total were 0.22 (0.00–1.08), 0.97 (0.28–3.76), 0.45 (0.15–1.40), and 3.44(1.24–8.04), respectively. ILI + rates showed predictable periodic fluctuations (Fig. 2). The rates of ILI + A/H1N1 and ILI + B exhibited a distinct peak in winter/spring with less pronounced activity in summer and autumn. There were two seasonal peaks of ILI + A/H3N2 rate and ILI + total rate, occurring in the winter-spring convergence season and in the summer. Kendall’s tau-b (τ_B_) correlation coefficients between independent variables of interest are listed (Table 2). Relative humidity was moderately correlated with total rainfall (τ_B_ = 0.464).

**Table 1:** Descriptive statistics of meteorological variables, air pollutants, and ILI + rates over 1998–2019

	5th percentile	25th percentile	Median	75th percentile	95th percentile
Meteorological variables					
Mean temperature (°C)	15.08	19.50	24.61	27.97	29.70
Mean relative humidity (%)	62.79	74.43	79.64	83.57	88.71
Total rainfall (mm)	0.00	0.30	11.35	57.50	204.95
Actual vapour pressure (hPa)	12.09	17.46	24.46	30.99	32.89
Air pollutants					
O_x_ (µg/m^3^)	22.78	32.78	42.78	53.11	66.00
SO_2_ (µg/m^3^)	5.60	9.13	13.15	18.60	27.85
PM_2.5_ (µg/m^3^)	10.93	20.56	30.34	42.75	62.35
Weekly ILI + rates (per 1000 admissions)					
ILI + A/H1N1	0.00	0.00	0.22	1.08	7.07
ILI + A/H3N2	0.00	0.28	0.97	3.76	14.12
ILI + B	0.00	0.15	0.45	1.40	5.13
ILI + total	0.38	1.24	3.44	8.04	20.05

ILI +, influenza-like illness-positive; O_x_, redox-weighted oxidant capacity; SO_2_, sulfur dioxide; PM_2.5_, fine particulate matter

**Table 2:** Kendall’s tau-b (τ_B_) correlation coefficients between environmental variables and ILI + rates

	Air Temperature	Relative Humidity	Total rainfall	Actual vapour pressure	O_x_	SO_2_	PM_2.5_	ILI + A/H1N1	ILI + A/H3N2	ILI + B	ILI + total
Air Temperature		0.102^**^	0.286^**^	0.803^**^	−0.224^**^	−0.007	−0.387^**^	−0.110^**^	0.091^**^	−0.087^**^	−0.036
Relative Humidity			0.464^**^	0.300^**^	−0.372^**^	−0.132^**^	−0.354^**^	0.106^**^	0.113^**^	0.131^**^	0.185^**^
Total rainfall				0.417^**^	−0.337^**^	−0.081^**^	−0.386^**^	0.016	0.096^**^	0.020	0.075^**^
Actual vapour pressure					−0.329^**^	−0.047^*^	−0.474^**^	−0.067^**^	0.116^**^	−0.042^*^	0.021
O_x_						−0.013	0.451^**^	−0.026	−0.137^**^	−0.081^**^	−0.156^**^
SO_2_							0.364^**^	−0.090^**^	0.074^**^	0.086^**^	0.065^**^
PM_2.5_								−0.063^**^	−0.078^**^	0.013	−0.050^*^
ILI + A/H1N1									−0.076^**^	0.192^**^	0.293^**^
ILI + A/H3N2										0.108^**^	0.551^**^
ILI + B											0.380^**^

ILI+, influenza-like illness-positive; O_x_, redox-weighted oxidant capacity; SO_2_, sulfur dioxide; PM_2.5_, fine particulate matter

*. Correlation is significant at the 0.05 level (2-tailed)

**. Correlation is significant at the 0.01 level (2-tailed)

**Fig. 3:**
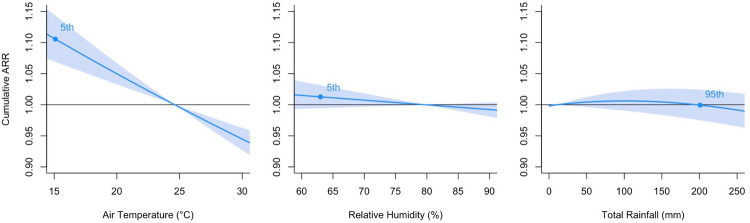
Cumulative ARRs on stroke admissions against different meteorological variables from 1998 to 2019. The reference values were set as medians for air temperature and relative humidity, and zero for total rainfall. The degrees of freedom were all equal to 5. The cumulative adjusted relative risks are expressed with 95% confidence intervals

Among the meteorological covariates, low air temperature was statistically significantly associated with an increased rate of stroke admission (Fig. 3). Compared with the median reference level (i.e., 24.61 °C), the cumulative ARR of stroke admissions was 1.106 (95% CI, 1.069–1.143) at the 5th percentile (i.e., 15.05 °C) of air temperature. Relative humidity and total rainfall were not associated with the stroke admissions. A similar insignificant trend was showed when relative humidity was replaced with actual vapour pressure (Supplementary Figure S1).

**Fig. 4:**
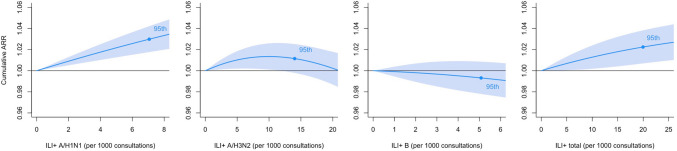
Cumulative ARRs on stroke admissions against different ILI + rates from 1998 to 2019. The reference values were set as zero for all ILI + rates. The degrees of freedom were all equal to 5. The cumulative adjusted relative risks are expressed with 95% confidence intervals

After adjustment for meteorological factors and air pollutants, the cumulative ARR of stroke admission increased monotonically with the rise in the ILI + A/H1N1 rate and the ILI + total rate, whereas the ILI + A/H3N2 rate and the ILI + B rate were not associated with stroke admissions (Fig. 4). Using zero as the reference, the cumulative ARRs were increased to 1.030 (95% CI, 1.018–1.042), and 1.022 (95% CI, 1.007–1.038) at the 95th percentile of ILI + A/H1N1 and ILI + total, respectively. The results of the model were similar without accounting for the potential influence of meteorological variables and air pollutants (Table 3).

**Table 3:** Adjusted relative risk of stroke at 95th percentile by different lag times of ILI + rates

	ILI + A/H1N1	ILI + A/H3N2	ILI + B	ILI + total
With adjusting meteorological variables and air pollutants				
*Lag zero*	1.004 (0.973–1.035)	0.997 (0.962–1.034)	0.991 (0.958–1.024)	0.989 (0.954–1.024)
*Lag at first week*	0.989 (0.943–1.038)	1.001 (0.953–1.053)	1.006 (0.966–1.047)	1.010 (0.960–1.062)
*Lag at second week*	**1.037 (1.006–1.069)**	1.012 (0.977–1.049)	0.997 (0.966–1.030)	1.024 (0.989–1.060)
*Overall*	**1.030 (1.018–1.042)**	1.011 (0.998–1.025)	0.993 (0.978–1.008)	**1.022 (1.007–1.038)**
Without adjusting meteorological variables and air pollutants				
*Lag zero*	1.011 (0.980–1.043)	1.004 (0.968–1.041)	0.993 (0.960–1.026)	0.996 (0.962–1.032)
*Lag at first week*	0.986 (0.940–1.035)	0.996 (0.946–1.048)	1.012 (0.972–1.055)	1.009 (0.959–1.062)
*Lag at second week*	**1.038 (1.006–1.070)**	1.009 (0.973–1.046)	0.995 (0.962–1.029)	1.020 (0.985–1.057)
*Overall*	**1.035 (1.022–1.047)**	1.009 (0.995–1.023)	1.000 (0.984–1.015)	**1.026 (1.010–1.042)**

ILI+, influenza-like illness-positive; **Bold**, statistical significance at *p*- value < 0.05. The reference level of ILI + rates was set as zero

**Fig. 5:**
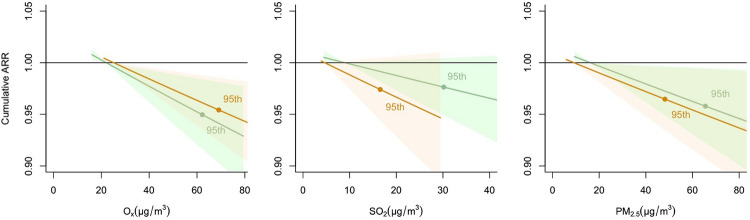
Cumulative ARRs on stroke admissions against different air pollutants stratified by year The cumulative ARRs in group from 1998 to 2010 are expressed as green color, and in group from 2011 to 2019 are expressed as orange color. The cumulative adjusted relative risks are expressed with 95% confidence intervals

In the stratified analyses (Fig. 5), SO_2_ was not significantly associated with stroke admissions, while O_x_ and PM_2.5_ were negatively associated with stroke admissions. Using the 5th percentile as the reference value, the cumulative ARR was decreased to 0.950 (95% CI, 0.916–0.984) for the 95th percentile (i.e., 62.20 µg/m^3^) of O_x_ from 1998 to 2010. Similarly, the cumulative ARRs at the 95th percentile of O_x_ from 2011 to 2019, 95th percentile of PM_2.5_ from 1998 to 2010, and 95th percentile of PM_2.5_ from 2011 to 2019 were 0.954 (95% CI, 0.923–0.985), 0.958 (95% CI, 0.922–0.995), and 0.964 (95% CI, 0.934–0.996), respectively.

## Discussion

This is a retrospective study based on population-based data encompassing most Hong Kong residents, with the objective of examining the association between meteorological factors, air pollutants, influenza infection, and stroke admissions. The results of our study indicated that there was an association between stroke admissions and cold weather, which is consistent with the results of previous systematic reviews (Lian et al. 2015; Wen et al. 2023). Studies from different regions have consistently shown that cold weather increases the relative risk of stroke. Studies in North America and Europe have shown that the risk of stroke can be increased by up to 1.2 times during cold spells (Gill et al. 2013; Jansen et al. 2001). Studies in Asia have shown that exposure to low temperatures ( < − 9.6 °C) can increase the risk of stroke by 1.3 to 1.5 times (Luo et al. 2018; Zeka et al. 2014). Several possible mechanisms have been proposed to explain cold weather as a trigger for stroke. Exposure to cold weather activates the sympathetic nervous system, increases platelet counts, and raises blood pressure, which can lead to blood clots and blood vessel ruptures, increasing the risk of ischemic and hemorrhagic stroke (Chambers et al. 2000; Hong et al. 2012; Liu et al. 2024; Ryti et al. 2017). In addition, cold weather triggers vasoconstriction and reduced peripheral circulation, while increased urination leads to blood concentration and hyperviscosity, which can increase the risk of hemorrhagic stroke and ischemic stroke (Liu et al. 2015; Neild et al. 1994; Wen et al. 2023). Furthermore, cold temperatures can increase the forces that contribute to the deformation of the vessel wall and the generation of frictional and shear stresses on the inner surface of the vessel, which are the trigger of hemorrhagic stroke (Vitale et al. 2015; Wen et al. 2023). According to existing studies, geographical latitude, atmospheric pressure, geomagnetic solar activity, GDP per capita, and gender may also have a modifying effect on the temperature-stroke relationship (Alahmad et al. 2023; Zorrilla-Vaca et al. 2017).

Furthermore, our study found that O_x_ was negatively associated with stroke admissions, which is consistent with the results of the European study of Cohorts for Air Pollution Effects (de Bont et al. 2023; Wolf et al. 2021). Previous research has suggested that the bactericidal characteristics and effects on host defense of O_3_ lead to a reduction in influenza transmission (Zhang et al. 2022), which in turn leads to a reduction in stroke admissions. Another more plausible mechanism is that inhalation of ambient O_3_ enhances pulmonary immunity to influenza virus infection (Ali et al. 2018). Some studies have shown that the negative association between O_3_ and stroke may be due to the negative association of other air pollutants, such as nitric oxide and ethylbenzene (Männistö et al. 2015), which were not included in our study and were more strongly associated with cardiovascular events. Previous studies on the association between NO_2_ and stroke have shown inconsistent results. A cross-sectional study in Shenzhen found that NO_2_ was positively associated with the risk of ischemic stroke hospitalizations throughout the year (Li et al. 2022). However, some studies have shown no association between exposure to nitrogen dioxide and the risk of stroke, possibly because of differences in the models used to assess exposure (Butland et al. 2017; Stafoggia et al.^2014^). In addition, this study found that high PM_2.5_ concentrations were negatively associated with stroke, which was consistent with the findings of the Swedish administrative groups (de Bont et al. 2023). They suggested that there were geographical differences in PM_2.5_ concentrations and stroke incidence that were not fully captured by the covariates included in the adjustment (de Bont et al. 2023). Furthermore, the present study found that SO_2_ was not associated with stroke admission, which is inconsistent with previous findings. Both a case-crossover study and a meta-analysis demonstrated a positive association between SO_2_ concentrations and the incidence of stroke (Liu et al. 2017; Shah et al. 2015). This discrepancy may be attributed to the strong association between SO_2_ and ischemic stroke admissions, in contrast to a relatively weak association with hemorrhagic stroke (Shen et al. 2020).

Influenza infection, particularly influenza A/H1N1, was associated with the risk of stroke hospitalizations at the population level. The results of the present study are consistent with those of a case-crossover study that demonstrated an increased risk of stroke within 15 days of ILI+, which can be regarded as a potential trigger for stroke (Boehme et al. 2018). Previous study suggested that influenza infection may act as a trigger for myocardial infarction and cardiovascular death (Warren-Gash et al. 2009). In addition, systemic infections such as influenza may induce an immune response through a procoagulant state, thereby increasing the risk of stroke (Hansson and Nilsson 2009; Zahhar et al. 2023). Nevertheless, the precise mechanism by which ILI causes stroke remains unclear, and further research is required to elucidate the potential causal relationship between ILI and stroke.

While a Japanese study showed that a low temperature and humidity change were related to stroke occurrence (Matsumaru et al. 2020), our study does not show a significant relationship between relative humidity and stroke admissions. In fact, the mechanism underlying the association between relative humidity and stroke risk remains unclear. Humidity changes are suggested to affect blood pressure regulation, potentially increasing the risk of stroke occurrence, as evidenced by findings that extreme humidity levels are associated with cerebrovascular hospitalizations (Doi et al. 2023). We acknowledge that our study does not control for the seasonality of relative humidity which may relate to temperature in influencing the stroke hospitalizations. Nevertheless, absolute humidity, a humidity metric independent to the temperature changes, was used in the analysis, and the results remained robust although a larger degree of uncertainty was observed. Since the role of humidity in influencing stroke is generally weaker than that of temperature, this may explain the insignificant relationship observed in our study.

A significant strength of this study is the completeness of the data, as our study analyzed admission data from all public hospitals in Hong Kong from 1998 to 2019. Over 1.1 million stroke-related admissions were recorded during this 22-year span. Moreover, previous studies have not elucidated the effects of meteorological variables, air pollutants, and influenza infection on stroke hospitalizations. Therefore, these findings may inspire future higher-quality studies in this area, such as a prospective cohort study considering all the influential factors identified in this study.

The present study has several limitations. First, we were unable to match the ILI + rates in different age and gender subgroups due to the lack of individual data on patients with influenza. Second, the potential impact of influenza vaccination rates was not considered in the statistical analyses, despite the recognition of a low vaccination rate in Hong Kong during the study span (Chan et al. 2015). Third, as the diagnosis of stroke was based solely on ICD classification, it is prone to misdiagnosis and underdiagnosis. Fourth, as this is a modelling study that employed pooled data to draw conclusions about associations of interest, ecological fallacies may occur. Fifth, we were unable to distinguish between ischemic stroke and hemorrhagic stroke. Given the potential for differing underlying mechanisms between different types of strokes, it is plausible that the effects of environmental exposure may also vary. Sixth, we used the admission rate of stroke as the outcome in the models, with the denominator potentially influenced by the number of other admissions. While we included week and year terms to adjust for variations over time, using rates is preferred to account for fluctuations, especially since our study aimed to examine whether the risk of stroke admissions is increasing or decreasing relative to the population.

## Conclusion

This study found that cold weather and influenza infection were associated with stroke admissions among adults in Hong Kong. Strategies for stroke prevention should target high-risk populations with a focus on cold weather conditions. Influenza vaccination programmes during winter deserve attention, not only to reduce influenza infection but also to reduce the risk of stroke. In addition, our findings have the potential to enhance the planning of healthcare resources. For instance, contingency plans could be devised to cope with the increase in hospital admissions during the winter months since more patients would arrive.

## Supplementary Information

Below is the link to the electronic supplementary material.

ESM 1 (JPG 1.51 MB)

## Data Availability

The data that support the findings of this study are available from the Hospital Authority, The Government of the Hong Kong Special Administrative Region but restrictions apply to the availability of these data, which were used under license for the current study, and so are not publicly available. Data are however available from the authors upon reasonable request and with permission of the Hospital Authority.

## Abbreviations

ARR: adjusted relative risk
CI: confidence interval
DLNM: distributed-lag non-linear model
EPD: Environmental protection department
ICD-9-CM: International classification of diseases
ILI: influenza-like illness
NO_2_: nitrogen dioxide
O_3_: ozone
PM_2.5_: fine particulate matter
SO_2_: sulfur dioxide
O_x_: oxidation capacity
VLM: value of lost welfare
